# Post-Embryonic Transcriptomes of the Prawn *Macrobrachium rosenbergii:* Multigenic Succession through Metamorphosis

**DOI:** 10.1371/journal.pone.0055322

**Published:** 2013-01-25

**Authors:** Tomer Ventura, Rivka Manor, Eliahu D. Aflalo, Vered Chalifa-Caspi, Simy Weil, Omri Sharabi, Amir Sagi

**Affiliations:** 1 Department of Life Sciences, Ben-Gurion University of the Negev, Beer-Sheva, Israel; 2 The National Institute for Biotechnology in the Negev, Ben-Gurion University of the Negev, Beer-Sheva, Israel; Academia Sinica, Taiwan

## Abstract

Like many metazoans, the freshwater prawn *Macrobrachium rosenbergii* begins its post-embryonic life with a set of morphologically distinct planktonic larval stages, followed by a benthic post-larval stage during which the maturing organism differs from the larvae both ecologically and physiologically. Understanding of the molecular basis underlying morphogenesis in crustaceans is limited to the observation that methyl farnesoate, the non-epoxidated form of the insect juvenile hormone, acts as the active crustacean juvenoid. Molt steroids were also linked to morphogenesis and several other molecular pathways, such as Hedgehog and Wnt, are known to underlie morphogenesis in all metazoans examined and, as such, are thought to do the same in crustaceans. Using next generation sequencing, we deep-sequenced the transcriptomes of several larval and post-larval stages. *De novo* assembly, followed by bioinformatics analysis, revealed that many novel transcripts are over-expressed in either larvae- or post-larvae-stage prawn, shedding light on the molecular basis underlying *M. rosenbergii* metamorphosis. Fast larval molting rates and periodic morphological changes were reflected in over-expression of transcripts annotated to the cell cycle, DNA replication and morphogenic pathways (i.e., Hedgehog and Wnt). Further characterization of transcripts assigned to morphogenic pathways by real-time RT-PCR reconfirmed their over-expression in larvae, albeit with a more complex expression pattern when examined in the individual developmental stages. The expression level of an orthologue of cytochrome P450, 15A1, known to epoxidize methyl farnesoate in insects, was increased in the late larval and early post-larval stages, in accordance with the role of methyl farnesoate in crustacean metamorphosis. This study exemplifies the applicability of a high-throughput sequencing approach for studying complex traits, including metamorphosis, providing new insight into this unexplored area of crustacean research.

## Introduction

Metamorphosis refers to the set of drastic post-embryonic anatomical and physiological changes that occur mostly in arthropods and amphibians when an immature individual transforms into an adult, and is usually accompanied by a change of habitat and/or behavior [1]. A common feature of metamorphosis in the animal kingdom is a biphasic life cycle where pelagic larvae metamorphose into benthic adults. This pelago-benthic transition, that also occurs in crustaceans such as the study organism, *Macrobrachium rosenbergii*, is orchestrated by numerous factors.

Presently, knowledge regarding the molecular basis underlying differences between crustacean larvae and post-larvae (PLs) is scarce. Apart from the fundamental understanding that 20-hydroxy ecdysone is the key molting regulator and that methyl farnesoate (MF), the non-epoxidated form of the insect juvenile hormone (JH III), governs the metamorphic transition [2], [3], little is known. In insects, farnesoic acid is converted to MF by farnesoic acid methyltransferase (FAMeT). MF is then epoxidated by CYP15A1 in the *corpora allata*, yielding JH III [4], the active juvenile hormone in insects [5]. JH III has yet to be detected in crustaceans, while MF, produced and secreted by the mandibular organ, is considered to be the juvenile hormone in this group [2]. MF was previously identified in *M. rosenbergii* [6], with its administration prolonging late larval stages [7], further strengthening the notion of MF being the crustacean juvenile hormone.

The intertwining Hedgehog and Wnt signaling pathways are known to be involved in morphogenesis in many metazoan groups [8], [9]. Their involvement in crustacean metamorphosis has, however, yet to be addressed. In recent morphogenic transcriptomic analysis of a marine bryzoan ancestrula, varying spatio-temporal patterns of Wnt signaling pathway component expression was reported, implicating this pathway in polypide patterning [10].

The use of high-throughput transcriptomics of animals at key developmental stages enables an unfolding of the changes occurring in transcription activity in relation to the morphological, anatomical and ecological states of the organism. Indeed, high-throughput transcriptomics is well suited for studying traits as complex and multi-systemic as those traits that accompany metamorphosis. Genome-wide transcriptional changes that occur during the shift from the pelagic to the benthic phases has been studied in only few animals, including some *Porifera*, ascidians, gastropods and corals [11]–[17]. In arthropods, the transcriptomic changes that transpire throughout the metamorphic life stages have been examined in only a few hexapod species (e.g. [18], [19]) and more recently, in the amphipod *Parhyale hawaiensis* [20] and the barnacle *Balanus amphitrite* [21]. Many transcriptomic datasets of the economically important group of decapod crustaceans are based upon Sanger sequencing of cDNA libraries (reviewed by Zeng *et al*., [20]. Today, next generation sequencing technologies that are revolutionizing genetic studies [22] are becoming increasingly affordable, accessible and robust even for organisms lacking a sequenced genome. Recently, comprehensive transcriptomes of adult tissues were obtained using 454 pyrosequencing for two of the most economically important freshwater prawn species, *M. rosenbergii* [23] and *M. nipponense* [24]. Still, larval and juvenile transcript expression patterns in decapods, both before and after metamorphosis, have yet to be investigated on a large-scale.

Like other *Macrobrachium* prawns, *M. rosenbergii* is a eurohaline species where egg-berried females migrate downstream in rivers to estuaries to reach elevated salinity that is crucial for larvae survival during the extended planktonic larval stage of development. After metamorphosing through 11 planktonic larval stages (termed zoea 1–11) that are morphologically different from one another [25], the benthic PLs that emerge are basically growing juveniles, capable of osmo-regulation in freshwater. Mature PLs then migrate up-stream in the river to join the older, hierarchical populations [26].

In this study, we employed a high-throughput next generation sequencing technique with the aim of characterizing key factors involved in metamorphosis in *M. rosenbergii*. By comparing the larval and post-larval transcriptomes generated, we identified important genes implicated in metamorphosis, including key components of the Hedgehog and Wnt signaling pathways, as well as components involved in MF metabolism. Numerous novel transcripts were found to be differentially expressed between larvae and PLs and were annotated with onthologies that were not connected to metamorphosis prior to this study.

## Results and Discussion

### Transcriptome Profiling

**Figure 1:**
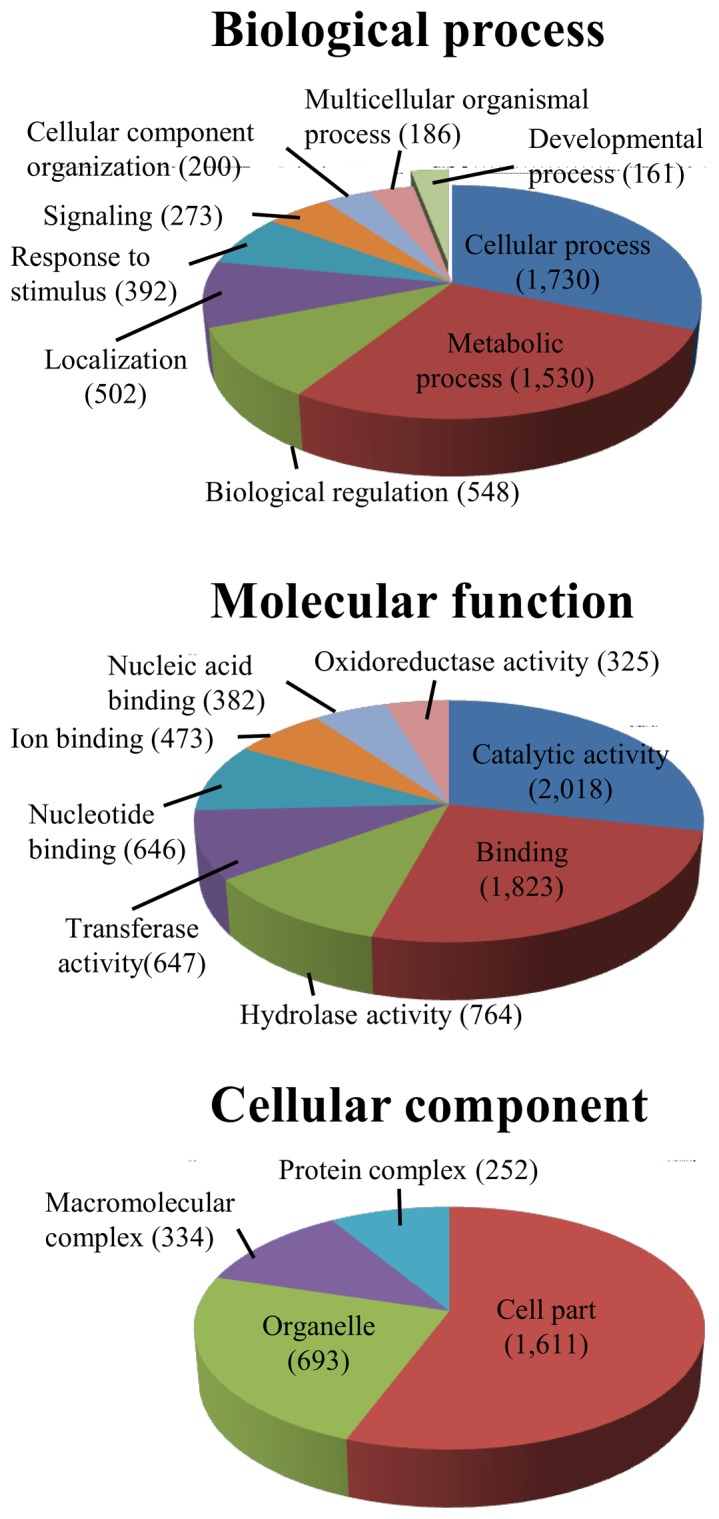
Most prominent GO terms assigned to the *M. rosebergii* developmental transcriptome. The most prominent GO terms assigned to level 2 of biological processes (top) and levels 2 and 3 of molecular functions (middle) and cellular components (bottom) are listed, as calculated by Blast2GO software suite. A small fraction of 161 GO terms (∼1.4%) are assigned to developmental processes.

One hundred bp-paired end sequencing in a single lane by an Illumina Genome Analyzer (HighSeq 2000, Illumina, San Diego, CA) generated ∼242 million reads from the cDNA of 200 *M. rosenbergii* larvae (zoea 4–11, 4 pooled samples) and PL_2–28_ (2–28 day post-metamorphosis, 4 pooled samples). *De novo* assembly using Velvet (K-mer 83) followed by Oases produced 66,152 Oases transcripts (contigs) with N50 = 1,651 bp and an average transcript length of 921 bp. As compared with the recently reported *M. rosenbergii* adult tissues transcriptome [23], our transcriptome is much more comprehensive, with 24.2 Gbp sequenced in total, yielding more transcripts (66,152, as compared with 8,411) of higher average length (921, as compared with 845, see Fig. S1 for length and coverage distribution). 11,528 Gene onthology (GO) terms were assigned to 7,076 transcripts, of which 4,011 GO terms were assigned biological processes, 5,172 were assigned molecular functions and 2,345 were assigned cellular components (Fig. S2). The most prominent level 2 GO terms for biological processes and levels 2 and 3 for molecular functions and cellular components are presented in Fig. 1. A small fraction of GO terms (∼1.4%) was assigned to developmental processes (161; Fig. 1).

A total of 54,073 transcripts yielded at least one BLASTX hit (against the UniRef90 database), of which 17,733 transcripts provided at least one hit with an e-value <10^−5^, an alignment length ≥50 amino acids and an alignment region cover >50% of the transcript length. 13,126 transcripts met similar conditions, with the exception of an alignment length of ≥100 amino acids. These transcripts were termed *Annotated transcripts*.

### Developmental transcriptome analysis

The coverage of each transcript (i.e. the number of reads mapped to it) in each sample was computed and further normalized to reads per million (RPM) values. Table 1 summarizes the number of *Annotated transcripts* found to be differentially expressed (>2-fold mean expression in PL/larvae or *vice versa*) in the *M. rosenbergii* developmental transcriptome database, with and without a *P*-value ≤0.05 cut-off. The ratios in Table 1 represent the number of transcripts over-expressed in larvae divided by the number of transcripts over-expressed in PLs. This ratio favors larvae, with ∼2.8-fold more transcripts being over-expressed in larvae than are over-expressed in PLs. This result is in keeping with the recent genome-wide analysis of the pelago-benthic transition phases of the sponge *Amphimedon queenslandica*, where ∼2-fold more transcripts were shown to be over-expressed in the free-swimming larvae [11]. A list of transcripts (>100) with the highest-fold change (with twice as many transcripts being over-expressed in larvae) is given in Fig. S3 (where either at the larval or post-larval group, the expression in each replicate is at least RPM = 5).

**Table 1:** Number of transcripts differentially represented in the *M. rosenbergii* developmental transcriptomic database.

Number of transcripts		
Fold-change ≥2	Fold-change ≥2 *P*-value ≤0.05	Stage
3,704	1,939	Larvae
1,281	697	Post-larvae
2.89	2.78	Ratio (Larva/Post-larvae)

**Figure 2:**
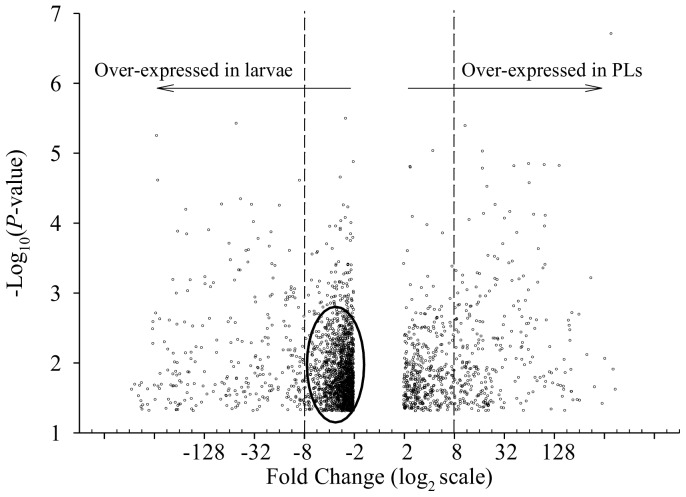
Volcano plot of transcript expression differences between larval and post-larval prawns. The magnitude of the difference in gene expression between larval and post-larval *M. rosenbergii* is shown on the X-axis on a log_2_ scale (post-larval/larval) of intensity (with minimum 2-fold change). The significance of the difference is given as the -log_10_ (*P*-value) obtained from a t test (with a minimum *P*-value  = 0.05). The mass of transcripts that are slightly over-represented in larvae (2–8-fold) are circled. Dashed lines represent the border of 8-fold change in larvae (-8 and left) and post-larvae (8 and right).

The differential expression measures of transcripts with at least 2-fold change between larvae and PLs and *P*-value ≤0.05 (Table 1) are represented in a volcano plot (Fig. 2). Negative values on the X-axis correspond with the 1,939 transcripts over-expressed in larvae, while the positive values correspond with the 697 transcripts over-expressed in PLs (with numbers corresponding to fold change). The majority of transcripts over-expressed in larvae fall within the 2- to 8-fold change region (dense area, circled in Fig. 2). When comparing the number of transcripts over-expressed by 8-fold or more (Fig. 2, dots outside the range between the dashed lines), only 270 transcripts are detected in larvae and 286 are detected in PLs.

### KEGG analysis

**Figure 3:**
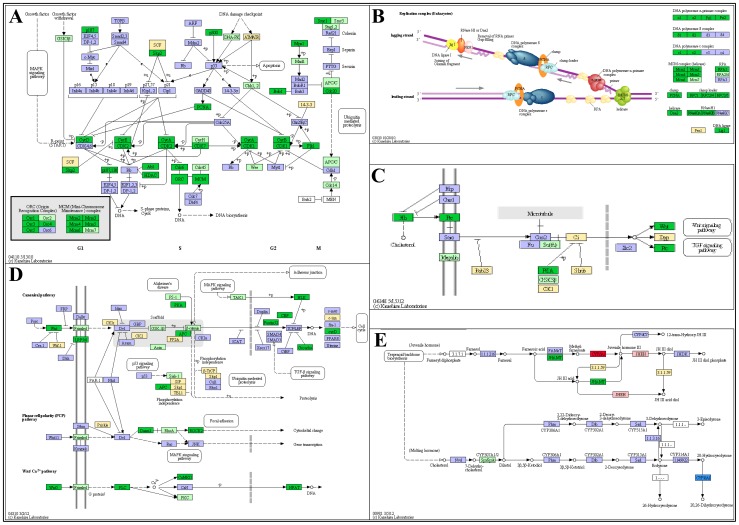
Representative pathways enriched in larvae or relative to metamorphosis. Cell cycle (**A**) and DNA replication (**B**) pathways, Hedgehog (**C**) and Wnt (**D**) signaling pathways all have higher representation in larvae than in PLs, while in the insect hormone synthesis pathway (**E**), the expression pattern is more ambiguous. KO terms present in KEGG pathways but absent in the *M. rosenbergii* developmental transcriptome are highlighted in purple. KO terms with ≥2-fold over-expression in larvae are in green, and in pale green when the ratio falls between 1.5 and 2. Yellow denotes KO terms found in our transcriptome showing no differential representation between larvae and PLs. Red denotes KO terms with ≥2-fold over-expression in PLs, changing to pink when the ratio falls between 1.5 and 2. Blue denotes ambiguous expression between larvae and PLs. Hedgehog and Wnt signaling pathways include Patched receptor (Ptc), Wnt5 and Frizzled, whose encoding transcripts were validated using real-time RT-PCR (**C** and **D**).

To map transcripts onto universal pathways, Oases transcripts were submitted to KAAS (see Materials and Methods for details). 3,873 transcripts were assigned 1,853 KEGG Orthology (KO) terms (with 2,020 redundant KO terms). The most prominent KEGG pathways represented in our transcriptome were metabolic pathways (n = 604), biosynthesis of secondary metabolites (n = 172), RNA transport (n = 114), pathways in cancer (n = 111), protein processing in endoplasmic reticulum (n = 102) and the spliceosome (n = 101). As an example of the higher percentage of transcripts over-expressed in larvae, the KEGG cell cycle (Fig. 3A) and DNA replication (Fig. 3B) pathways are color coded, where green represents a larvae/PL fold change ≥2 and pale green represents a larvae/PL fold change between 1.5 and 2. Overall, this data suggests that higher rates of mitotic activity may occur in *M. rosenbergii* larvae as compared with PLs, a phenomenon that might be attributed to the shorter molt intervals in larvae [25] and frequent morphological changes, or, alternatively, to the fact that the RNA content in larval stages is lower than in PLs, as shown with the white shrimp *Litopenaeus schmitti* [27]. However, this remains to be studied in depth, and will rely on a more refined dataset that examines each larval stage separately, in particular distinguishing between different molt stages. Nevertheless, our study highlights candidate genes to be tested in this regard.

The best represented KEGG signaling pathways in our transcriptome were the MAPK (n = 72), Wnt (n = 56), insulin (n = 53), chemokine (n = 43), neurotropin (n = 40), calcium (n = 36), epithelial cell signaling pathway in *Helicobacter pylori* infection (n = 31), ErbB (n = 30), GnRH (n = 30), TGF-beta (n = 30), phosphatidylinositol (n = 27), T-cell receptor (n = 26), mTOR (n = 25), p53 (n = 25), Jak-STAT (n = 21), adipocytokine (n = 21), Notch (n = 21), Toll-like receptor (n = 20), VEGF (n = 20), B-cell receptor (n = 18), PPAR (n = 18) and Hedgehog (n = 17) pathways. Interestingly, most transcripts assigned to the intertwining Hedgehog and Wnt signaling pathways, known to be involved in morphogenesis [8], [9], showed higher expression in larvae than in PLs (Fig. 3C, D). This could be due to the distinct morphological changes that occur over the course of the 11 planktonic larval stages [25], changes that do not take place during the post-larval stages. In the insect hormone synthesis pathway, only transcripts of 6 enzymes were identified, of which two showed higher expression in larvae and two others showed higher expression in PLs (Fig. 3E).

Table 2 summarizes the number of KO terms assigned to differentially expressed transcripts that are listed in Table 1, with and without a *P*-value ≤0.05 cut-off. Since several transcripts share the same KO term, the number of unique KO terms is also included in Table 2. The ratios in Table 2 represent the number of KO terms assigned to transcripts with higher expression in larvae than in PLs, divided by the number of KO terms assigned to transcripts with higher expression in PLs. As in Table 1, the ratio favors larvae, with ∼7- and ∼4.7-fold (unique and total, respectively) higher numbers of KO terms assigned to transcripts with higher expression in larvae than in PLs. When comparing the number of KO terms assigned to transcripts with 8-fold expression or higher with *P*-values ≤0.05 (270 transcripts in larvae and 286 in PLs), 34 KO terms were assigned to 65 transcripts with higher expression in larvae and 23 KO terms were assigned to 52 transcripts with higher expression in PLs. These factors include protein and lipid metabolism factors, in addition to a predicted Hedgehog receptor termed *Mr-Patched* (Fig. 3C, Ptc) that is ∼20 times higher expressed in larvae than in PLs.

**Table 2:** Number of KO terms assigned to differentially represented transcripts in the *M. rosenbergii* developmental transcriptomic database.

Number of KO terms				
Unique		Total		
Fold-change ≥2	Fold-change ≥2 *P*-value ≤0.05	Fold-change ≥2	Fold-change ≥2 *P*-value ≤0.05	Stage
807	534	1,172	726	Larvae
114	76	250	154	Post-larvae
7.08	7.03	4.69	4.71	Ratio (Larva/Post-larvae)

### Characterization of genes implicated in metamorphosis

The Hedgehog and Wnt pathways belong to the central canon of developmental signaling pathways in metazoans [28]. Hedgehog spatial-temporal expression patterns change throughout embryonic and post-embryonic development to determine cell fate and by doing so, defines tissue boundaries, left to right axial orientation and organogenesis [29], [30]. In *Drosophila*, Hedgehog spatial-temporal expression was shown to be crucial for segment polarity and cuticle patterning in larvae and adults [31]. Wnt5, a ligand of the Wnt signaling pathway, is a morphogen serving multiple functions during development, including planar cell polarity signaling pathway activation [32]. Homologous genes of *Patched* (*Mr-Patched*, 853 amino acids; Fig. 3C, Ptc), the Hedgehog receptor, a ligand, *Wnt5* (*Mr-Wnt5*, 339 amino acids; Fig. 3D, Wnt5), and *Frizzled* (*Mr-Fz*, 610 amino acids; Fig. 3D, frizzled), a receptor of the Wnt pathway, were characterized, along with *Mr-CYP15A1* (472 amino acids; Fig. 3E, CYP15A1), an ortholog of the insect MF-epoxidizing enzyme [4]. Orthology of *Mr-Wnt5* and *Mr-CYP15A1* was supported by phylogenetic analysis using the Maximum parsimony method (Fig. S4). *Mr-Patched* consists of 853 amino acids, encompassing a signal peptide and a Patched domain, while *Mr-Fz* consists of 610 amino acids, encompassing a signal peptide and two Frizzled domains, as identified by SMART (Fig. S4). Partial cDNA sequences were deposited in Genebank, and their NCBI accession numbers and BLASTP results are summarized in Fig. S5. In our transcriptome analysis, while several FAMeT homologs are ∼3-fold over-expressed in larvae, a cytochrome P450 homolog of CYP15A1 was not detected in larvae yet peaked in PLs (Fig. 3E). *Mr-Patched*, *Mr-Wnt5* and *Mr-Fz*, on the other hand, presented higher expression levels in larvae (∼20-, ∼2- and ∼2-fold, respectively).

### Expressional profiling of key metamorphosis genes

In a recent morphogenic transcriptomic analysis of spatio-temporal expression patterns of Wnt signaling pathway components in the marine bryzoan ancestrula, transcript levels were shown to vary, implicating this pathway in polypide patterning [10]. Since in our developmental transcriptomic database components of Wnt pathway and the upstream Hedgehog pathway were over-expressed in larvae, we used real-time RT-PCT to re-evaluate the relative transcript levels of *Mr-Patched*, *Mr-Wnt5*, and *Mr-Fz* (frizzled), key genes in these pathways.

**Figure 4:**
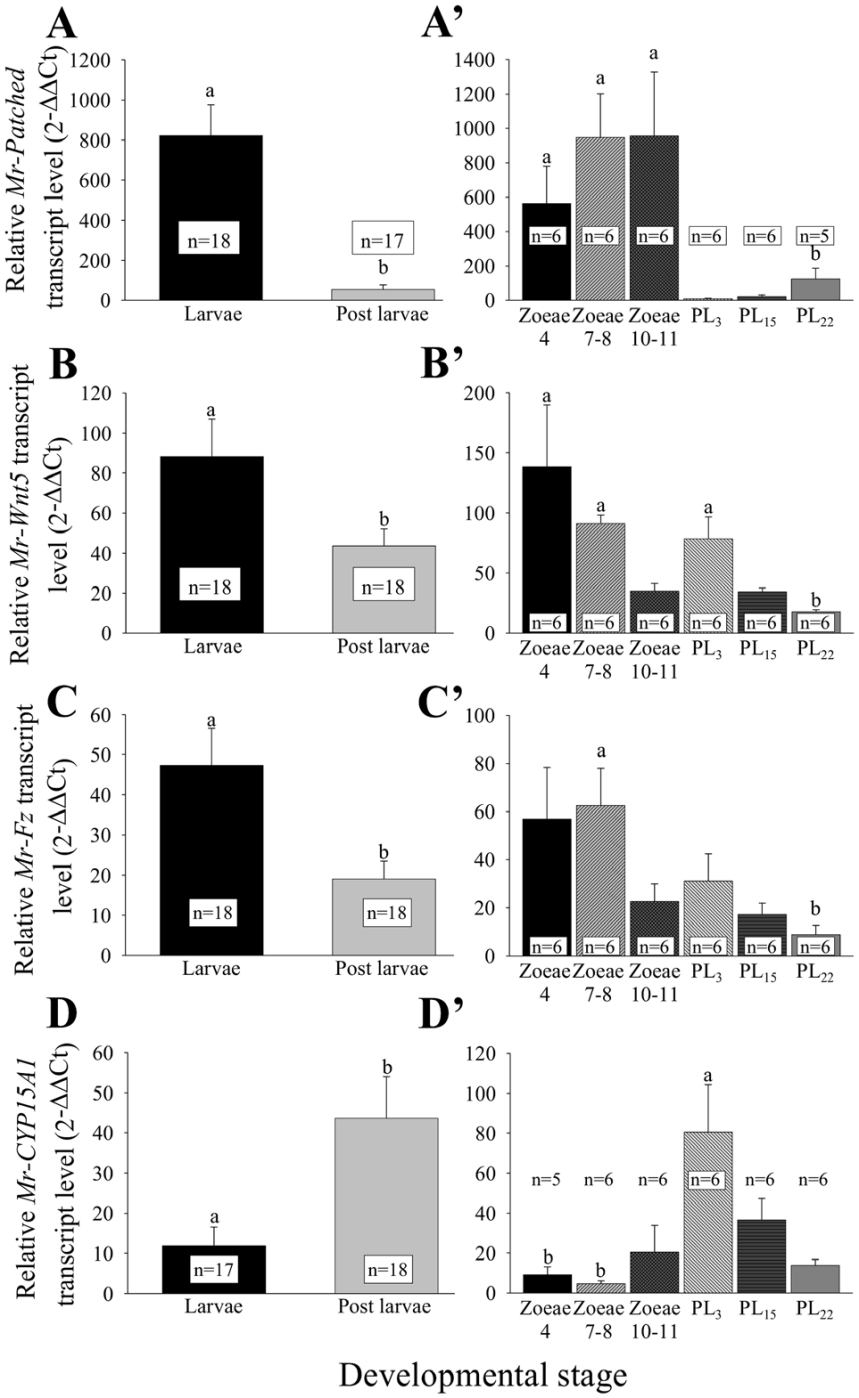
Relative transcript levels of key metamorphosis-related genes. Relative transcript levels of *Mr-Patched*, *Mr-Wnt5*, *Mr-Fz* and *Mr-CYP15A1* were quantified via real-time RT-PCR in larvae and PLs (n = 18 in each group). Different letters represent significant differences, while error bars represent SEM. Comparison of relative *Mr-Patched* (**A**), *Mr-Wnt5* (**B**) and *Mr-Fz* (**C**) transcript levels in larvae and PLs reveals a highly significant difference (Mann-Whitney U test, *P*-value <0.01), supporting the ratio of ∼20 (*Mr-Patched*) and ∼2 (*Mr-Wnt5* and *Mr-Fz*) in favor of the larval group compared with PLs, as calculated by the RPM ratio obtained with the transcriptome. Comparison of relative *Mr-CYP15A1* (**D**) transcript levels in larvae and PLs also reveals a highly significant difference (Mann-Whitney U test, *P*-value <0.01), supporting the higher expression levels expected in PLs, as calculated by the RPM ratio obtained with the transcriptome. However, when dividing the larval and PL groups into defined stages (Zoea 4, 7–8, 10–11, PL_3, 15, 22_), the overall trend is more complex, with significantly higher *Mr-Patched* transcript levels seen in Zoea 4, and 7–8, as compared with PL_15_ and PL_22_ and also in Zoea 10–11, as compared with PL_22_ (**A'**; Kruskal-Wallis test: H (df = 5, N = 36)  = 24.06317, *P*-value <0.05), significantly higher *Mr-Wnt5* transcript levels seen in Zoea 4, 7–8 and PL_3_, as compared with PL_22_ (**B'**; Kruskal-Wallis test: H (df = 5, N = 36)  = 28.50751, *P*-value <0.01), significantly higher *Mr-Fz* transcript levels in Zoea 7–8, as compared with PL_22_ (**C'**; Kruskal-Wallis test: H (df = 5, N = 36)  = 14.48048, *P*-value <0.02) and significantly higher *Mr-CYP15A1* transcript levels in PL_3_, as compared with Zoea 4 and Zoea 7–8 (**D'**; Kruskal-Wallis test: H (df = 5, N = 36)  = 17.66032, *P*-value <0.05).

Real-time RT-PCR reconfirmed that *Mr-Patched* is ∼20-fold over-expressed in larvae in our transcriptomic dataset. When comparing relative transcript levels of this gene in larvae and PLs (n = 18 in each group), a significant ∼20-fold higher expression level was observed in larvae (Fig. 4A, Mann-Whitney U test, *P*-value<0.01). When the larval and PL groups were divided into defined stages (Zoea 4, 7–8, 10–11, PL_3, 15, 22_; n = 6 in each group), significantly higher *Mr-Patched* transcript levels were observed in Zoea 4 and 7–8, as compared with PL_22_ and PL_15_, as well as in Zoea 10–11, as compared with PL_22_ (Fig. 4A', Kruskal-Wallis test: H (df = 5, N = 36)  = 24.06317, *P*-value <0.05).

*Mr-Wnt5* and *Mr-Fz* are both ∼2-fold over-expressed in larvae according to our transcriptomic dataset. This result was also confirmed via real-time RT-PCR. When comparing relative transcript levels of the markers in larvae and PLs (n = 18 in each group), a significantly higher level of ∼2-fold was observed in larvae (Fig. 4B, C, Mann-Whitney U test, *P*-value <0.01). However, when dividing the larval and PL groups into defined stages (Zoea 4, 7–8, 10–11, PL_3, 15, 22_; n = 6 in each group), the overall trend observed was more complex, with significantly higher *Mr-Wnt5* transcript levels in Zoea 4 and 7–8 and PL_3_, as compared with PL_22_ (Fig. 4B', Kruskal-Wallis test: H (df = 5, N = 36)  = 28.50751, *P*-value <0.01), and significantly higher *Mr-Fz* transcript levels in Zoea 7–8, as compared with PL_22_ (Fig. 4C', Kruskal-Wallis test: H (df = 5, N = 36)  = 14.48048, *P*-value <0.02).

*Mr-CYP15A1* was not detected in larvae and peaked in PLs in our transcriptome analysis. However, when examined via real-time RT-PCR, a slightly different picture appeared, with some expression being detected in larvae, albeit significantly lower than in PLs (Fig. 4D, Mann-Whitney U test, *P*-value <0.001). When dividing the larval and PL groups into defined stages (Zoea 4, 7–8, 10–11, PL_3, 15, 22_; n = 6 in each group), larval expression is mainly observed in the late larval stages (Zoea 10–11), with a significant higher relative transcript level being noted in PL_3_, as compared with Zoea 4 and Zoea 7–8 (Fig. 4D', Kruskal-Wallis test: H (df = 5, N = 36)  = 17.66032, *P*-value <0.05). Since CYP15A1 epoxidizes MF into JH III in insects [4] and MF is the active juvenile hormone in crustaceans [3], the fact that *Mr-CYP15A1* expression is elevated in late larval stages towards early PL stages suggests a mechanism for metamorphosis signal conveyed via metabolism of active MF by *Mr-CYP15A1* into the inactive form in *M. rosenbergii*.

## Conclusions

In this study, we assembled a comprehensive transcriptome of the early post-embryonic developmental stages of the giant freshwater prawn, *M. rosenbergii*. The accuracy of expression levels listed in this transcriptome was reconfirmed by real-time RT-PCR in the case of several key transcripts that are hypothesized to be involved in metamorphosis and indeed were found to be differentially transcribed in larvae and PLs. Interestingly, we found that the expression of a CYP15A1 ortholog is elevated in late larval and early PL stages. Since CYP15A1 is known to epoxidize MF (the crustacean juvenile hormone) into JH III (the insect juvenile hormone), it is plausible that the CYP15A1 ortholog is responsible for modifying MF, thereby reducing its levels, enabling metamorphosis.

Overall, we found that a higher number of transcripts are over-expressed in larvae, a phenomenon that could be attributed to the higher rates of cellular proliferative and differentiation activity of organisms in this stage, as phenotypically manifested by their intense molting, as well as by the morphological changes that accompany larval transitions [25]. Finally, we also highlighted numerous transcripts that can be considered as promising candidates for studies aimed at further elucidating the molecular mechanisms underlying metamorphosis in *M. rosenbergii*, based on the extreme shift in expression they undergo in either larvae or PLs.

## Materials and Methods

### Sample Preparation and Sequencing

Total RNA from *M. rosenbergii* larvae (Zoea 4 to 11) and PL_2–28_ (in several batches for each stage, with a total of 200 individuals) was isolated with the EZ-RNA Total RNA Isolation Kit, according to the manufacturer's instructions (Biological Industries, Beit Haemek, Israel). Equal amounts of RNA were pooled into four pools, each containing RNA from 40–60 individuals of 5 larval or PL sub-stages (8–15 individuals at each sub-stage), in duplicate. To ensure representation of low abundance transcripts in the transcriptome assembly, each of the four pools was divided into two tubes where one was subjected to enzymatic normalization. All eight samples were used for the *de novo* assembly, while differential expression was calculated only for the four non-normalized pools. These eight total samples were subjected to next generation sequencing by Fasteris Life Science (Geneva, Switzerland) on an Illumina Genome Analyzer (HighSeq 2000, Illumina, San Diego, CA), performing 100 bp–paired end sequencing in a single lane. The sequence reads were separated into 8 libraries according to their indexes (de-multiplexing) and stored as FASTQ files. This yielded a total of ∼242 million reads, with 14–38 (average 30) million reads per library. Specifically, the libraries included (in millions of reads): 26.6 and 38 in the larval duplicates, 37.4 and 34.6 in the PL duplicates, 26.2 and 14.6 in the normalized larval duplicates, and 28 and 36.4 in the normalized PL duplicates, respectively.

### *De novo* assembly and analysis

To create a reference transcriptome, all reads were *de novo* assembled using Velvet [33], with seven K-mer values (73, 79, 81, 83, 85, 87 and 95), followed by Oases [34]. The pair-read insert average size was set at 200 bases, with a standard deviation of 10%. To validate *de novo* assemblies, 1 million pairs of reads from each library were mapped to each assembly. The highest number of reads was mapped to the 81- and 83 K-mer assemblies. The latter was chosen as the preferred assembly, producing 66,152 Oases transcripts (contigs) with N50 = 1,651 bp and an average transcript length of 921 bp (with 58.2% to 70.5% of the reads assembled from each library). Functional annotation was carried out by conducting a BLASTX search of each transcript against the UniRef90 database. A total of 54,073 transcripts generated at least one BLASTX hit, of which 17,733 transcripts generated at least one hit with an e-value<10^−5^, an alignment length ≥50 amino acids and an alignment region cover >50% of the transcript length. 13,126 transcripts (termed *Annotated transcripts*) met similar conditions, except for having an alignment length ≥100 amino acids.

The Blast2GO software suite [35], [36] was used to predict transcript function and assign Gene Ontology terms [37], [38]. Blast2GO parameters included BLASTX against the GenBank non-redundant (nr) database hosted by the National Center for Biotechnology Information (NCBI) (http://www.ncbi.nlm.nih.gov/), with an e-value threshold <1e^–6^, retrieving 3 blast hits for each transcript. Mapping and annotation were performed using default parameters. Combined graphs were calculated with a minimum of 20 sequences per node filter. Transcript involvement in a metabolic pathway was predicted using the Kyoto Encyclopaedia of Genes and Genome (KEGG) server [39], [40]. The 66,152 Oases transcripts were submitted to KAAS (KEGG Automatic Annotation Server) as ‘Complete or Draft Genome’ using the bi-directional best-hit assignment method with all animal organisms selected.

### Differential expression analysis

For digital gene expression analysis, the reads from each library were separately mapped to the 83 K-mer Oases transcriptome using BWA. Reads that mapped to several positions on the reference with the same ‘mapping quality’ were randomly assigned to one of these. The coverage of each transcript (i.e. the number of reads mapped to it) was then computed using BEDtools software and further normalized to allow for comparison among libraries by dividing the number of mapped reads by the number of reads in the library ×1 million (abbreviated as RPM, reads per million). Since in subsequent analyses we considered only fold-change and *P*-value of transcript abundance differences between the developmental stages, and no comparisons were carried out among the transcripts themselves (nor their absolute expression levels were regarded), no further normalization to account for transcript length (a.k.a RPKM) was carried out. Comparison of larval and PL RPM values was carried out for each of the 13,126 *Annotated transcripts*, excluding the 4 normalized libraries. Fold-change values were obtained by dividing the mean RPM of the PL samples by the mean RPM of the larvae samples, after setting mean values lower than 0.05 to 0.05. Fold-change values lower than 1 were reformatted as -1/value. Transcripts showing two-fold change and more were considered as being differentially expressed. A t test was subsequently performed to identify transcripts with *P*-value <0.05.

### Gene characterization

Selected transcripts were translated into six reading frames using the ExPASy translate tool (http://web.expasy.org/translate/) and the most probable ORF was chosen in each case. By performing BLASTP searches against the GenBank non-redundant (nr) database hosted by NCBI, gene orthology assignments for selected ORFs were made. Reference ORF sequences from different organisms were downloaded from the NCBI protein sequence database and aligned with the deduced amino acid sequences of the corresponding *M. rosenbergii* ORFs using ClustalW [41], after which mismatches in the alignment were manually corrected. These alignments were then used for phylogenetic analysis in Mega, version 4.0 [42]. A maximum parsimony phylogenetic analysis using 500 bootstraps was calculated.

### Real-time RT-PCR

First-strand cDNA was synthesized by means of reverse transcriptase reaction using the Verso cDNA Kit (Thermo Fisher Scientific) with 1 µg of total RNA. Relative quantification of transcript levels was determined using the following primers with the FastStart Universal Probe Master (Rox, Roche Diagnostics) and Universal ProbeLibrary probes (Roche). For *Mr-Wnt5*, the following probes were used: q*Mr-Wnt5*_F: *5′-TTTCTGGGTCGTGCTCTCTC-3′* and q*Mr-Wnt5*_R: *5′-CGCCTACTTCCCTGAAAGGT-3′*, Probe #62; for *Mr-Fz*: q*Mr-Fz*_F: *5′-ATGCTCACGCCCTAACTCAG-3′* and q*Mr-Fz*_R: *5′-GTAGTTGTGGCAGTGGATGG-3′*, Probe #111; for *Mr-Patched*: q*Mr-Patched*_F: *5′-TCAAGACAAGCTTTCAATTTTCC -3′* and q*Mr-Patched*_R: *5′-ACAGCAAAGTCGTTGCGATA -3′*, Probe #92; for *Mr-CYP15A1*: q*Mr-CYP15A1*_F: *5′* -*CGTCATACTCGAGGAAGTCCA*-*3*′ and q*Mr-CYP15A1*_R: *5′* -*AAATTCGACGCTTCCATCAG*-*3*′. *Mr-18S* (accession no. GQ131934), which served as a normalizing gene, was also quantified by means of real-time RT-PCR using the primers: q*Mr-18S*_F: *5′-CCCTAAACGATGCTGACTAGC -3′* and q*Mr-18S*_R: *5′-TACCCCCGGAACTCAAAGA-3′* with the above-mentioned mix and Universal ProbeLibrary Probe #152 (Roche). Reactions were performed with the ABI Prism 7300 Sequence Detection System (Applied Biosystems, Foster City, CA).

### Statistical Analysis

The two groups examined by real-time RT-PCR were analyzed using the Mann-Whitney U test and multiple groups were analyzed using the Kruskal Wallis test, followed by the correction of a multiple pair-wise comparison (built-in within the STATISTICA software), as accepted.

## Supporting Information

Figure S1 Transcriptome profile. Distribution of contigs length (A) and normalized coverage (B).
(DOCX)

Figure S2 Gene Ontology (GO) annotations according to sequence. A list of all GO terms according to Blast2GO is given for all sequences which had at least one BLASTX hit with an e-value <10^−5^, an alignment length ≥100 amino acids and an alignment region cover >50% of the transcript length (the 13,126 termed *Annotated transcripts*). The full list is followed by smaller sub-lists according to biological processes, molecular functions and cellular components.
(XLSX)

Figure S3 Lists of contigs with >100-fold change over-expression in Larvae and PLs. Contig list was filtered to include transcripts with at least 500 bp and redundant transcripts of the same locus were not included.
(XLSX)

Figure S4 Bioinformatics characterization of four transcripts found to be correlated with metamorphosis. Partial transcript and deduced open reading frame is given for *Mr-Patched, Mr-Wnt5, Mr-Fz* and *Mr-CYP15A1*. Phylogenetic trees were generated by Mega 4.0 (alignment by ClustalW, maximum parsimony tree 500 bootstraps) for all four with representative orthologs from different taxonomic groups. SMART domain search in Mr-Patched yielded a signal peptide (red) and a Patched domain (grey) with high probability (e^−80^) and in Mr-Fz it yielded a signal peptide (red) and two Frizzled domains (orange and grey) with high probability (e^−66^).
(DOCX)

Figure S5 Best three BLASTP Results for *Mr-Patched, Mr-Wnt5, Mr-Fz* and *Mr-CYP15A1*.
(XLSX)

## References

[1] Medina M (2009) Functional genomics opens doors to understanding metamorphosis in nonmodel invertebrate organisms. Molecular Ecology 18: 763–764. 19207243 10.1111/j.1365-294X.2008.04079.x

[2] Laufer H, Biggers WJ (2001) Unifying concepts learned from methyl farnesoate for invertebrate reproduction and post-embryonic development. American Zoologist 41: 442–457.

[3] Laufer H, Borst D, Baker FC, Reuter CC, Tsai LW, et al (1987) Identification of a juvenile hormone-like compound in a crustacean. Science 235: 202–205. 17778635 10.1126/science.235.4785.202

[4] Helvig C, Koener JF, Unnithan GC, Feyereisen R (2004) CYP15A1, the cytochrome P450 that catalyzes epoxidation of methyl farnesoate to juvenile hormone III in cockroach corpora allata. Proceedings of the National Academy of Science of the United States of America 101: 4024–4029. 10.1073/pnas.0306980101 PMC384689 15024118

[5] Truman JW, Riddiford LM (1999) The origins of insect metamorphosis. Nature 401: 447–452. 10519548 10.1038/46737

[6] Sagi A, Homola E, Laufer H (1991) Methyl farnesoate in the prawn *Macrobrachium rosenbergii*: Synthesis by the mandibular organ *in vitro*, and titers in the hemolymph. Comparative Biochemistry and Physiology Part B: Comparative Biochemistry 99: 879–882. 10.1016/0305-0491(91)90157-9 1790679

[7] Abdu U, Takac P, Laufer H, Sagi A (1998) Effect of methyl farnesoate on late larval development and metamorphosis in the prawn *Macrobrachium rosenbergii* (Decapoda, Palaemonidae): A juvenoid-like effect? Biological Bulletin 195: 112–119. 28570170 10.2307/1542818

[8] Ingham PW, McMahon AP (2001) Hedgehog signaling in animal development: paradigms and principles. Genes & Development 15: 3059–3087. 11731473 10.1101/gad.938601

[9] Logan CY, Nusse R (2004) The Wnt signaling pathway in development and disease. Annual Review of Cell and Developmental Biology 20: 781–810. 10.1146/annurev.cellbio.20.010403.113126 15473860

[10] Wong YH, Wang H, Ravasi T, Qian PY (2012) Involvement of Wnt signaling pathways in the metamorphosis of the bryozoan *Bugula neritina* . PLoS ONE 7: e33323. 22448242 10.1371/journal.pone.0033323 PMC3308966

[11] Conaco C, Neveu P, Zhou H, Arcila ML, Degnan SM, et al (2012) Transcriptome profiling of the demosponge *Amphimedon queenslandica* reveals genome-wide events that accompany major life cycle transitions. BMC Genomics 13: 209. 22646746 10.1186/1471-2164-13-209 PMC3447736

[12] Azumi K, Sabau SV, Fujie M, Usami T, Koyanagi R, et al (2007) Gene expression profile during the life cycle of the urochordate *Ciona intestinalis* . Developmental Biology 308: 572–582. 17572404 10.1016/j.ydbio.2007.05.022

[13] Grasso LC, Maindonald J, Rudd S, Hayward DC, Saint R, et al (2008) Microarray analysis identifies candidate genes for key roles in coral development. BMC Genomics 9: 540. 19014561 10.1186/1471-2164-9-540 PMC2629781

[14] Grasso LC, Negri AP, Foret S, Saint R, Hayward DC, et al (2011) The biology of coral metamorphosis: molecular responses of larvae to inducers of settlement and metamorphosis. Developmental Biology 353: 411–419. 21338599 10.1016/j.ydbio.2011.02.010

[15] Heyland A, Vue Z, Voolstra CR, Medina M, Moroz LL (2011) Developmental transcriptome of *Aplysia californica* . Journal of Experimental Zoology 316B: 113–134. 21328528 10.1002/jez.b.21383 PMC4028319

[16] Reyes-Bermudez A, Desalvo MK, Voolstra CR, Sunagawa S, Szmant AM, et al (2009) Gene expression microarray analysis encompassing metamorphosis and the onset of calcification in the scleractinian coral *Montastraea faveolata* . Marine Genomics 2: 149–159. 21798184 10.1016/j.margen.2009.07.002

[17] Williams EA, Degnan BM, Gunter H, Jackson DJ, Woodcroft BJ, et al (2009) Widespread transcriptional changes pre-empt the critical pelagic-benthic transition in the vetigastropod *Haliotis asinina* . Molecular Ecology 18: 1006–1025. 19207244 10.1111/j.1365-294X.2008.04078.x

[18] Guerrero FD, Dowd SE, Sun Y, Saldivar L, Wiley GB, et al (2009) Microarray analysis of female- and larval-specific gene expression in the horn fly (*Diptera: Muscidae*). Journal of Medical Entomology 46: 257–270. 19351076 10.1603/033.046.0210

[19] Koutsos AC, Blass C, Meister S, Schmidt S, MacCallum RM, et al (2007) Life cycle transcriptome of the malaria mosquito *Anopheles gambiae* and comparison with the fruitfly *Drosophila melanogaster* . Proceedings of the National Academy of Science of the United States of America 104: 11304–11309. 10.1073/pnas.0703988104 PMC2040894 17563388

[20] Zeng V, Villanueva KE, Ewen-Campen BS, Alwes F, Browne WE, et al (2011) *De novo* assembly and characterization of a maternal and developmental transcriptome for the emerging model crustacean *Parhyale hawaiensis* . BMC Genomics 12: 581. 22118449 10.1186/1471-2164-12-581 PMC3282834

[21] Chen ZF, Matsumura K, Wang H, Arellano SM, Yan X, et al (2011) Toward an understanding of the molecular mechanisms of barnacle larval settlement: a comparative transcriptomic approach. PLoS ONE 6: e22913. 21829555 10.1371/journal.pone.0022913 PMC3146488

[22] Mardis ER (2008) The impact of next-generation sequencing technology on genetics. Trends in Genetics 24: 133–141. 18262675 10.1016/j.tig.2007.12.007 PMC2680276

[23] Jung H, Lyons RE, Dinh H, Hurwood DA, McWilliam S, et al (2011) Transcriptomics of a giant freshwater prawn (*Macrobrachium rosenbergii*): *de novo* assembly, annotation and marker discovery. PLoS ONE 6: e27938. 22174756 10.1371/journal.pone.0027938 PMC3234237

[24] Ma K, Qiu G, Feng J, Li J (2012) Transcriptome analysis of the oriental river prawn, *Macrobrachium nipponense* using 454 pyrosequencing for discovery of genes and markers. PLoS ONE 7: e39727. 22745820 10.1371/journal.pone.0039727 PMC3380025

[25] Uno Y, Chin Soo K (1969) Larval development of *Macrobrachium rosenbergii* (de Man) reared in the laboratory. Tokyo University of Fisheries 55: 179–190.

[26] FAO (2009) *Macrobrachium rosenbergii*: Species Fact Sheets. FAO.

[27] Lemos D, Garcia-Carreño FL, Hernández P, Navarrete del Toro A (2002) Ontogenetic variation in digestive proteinase activity, RNA and DNA content of larval and postlarval white shrimp *Litopenaeus schmitti* . Aquaculture 214: 363–380.

[28] Ingham PW, Nakano Y, Seger C (2011) Mechanisms and functions of Hedgehog signalling across the metazoa. Nature Reviews Genetics 12: 393–406. 10.1038/nrg2984 21502959

[29] Blaess S, Bodea G, Kabanova A, Chanet S, Mugniery E, et al (2011) Temporal-spatial changes in Sonic Hedgehog expression and signaling reveal different potentials of ventral mesencephalic progenitors to populate distinct ventral midbrain nuclei. Neural Development 6: 29 (19 pages).. 21689430 10.1186/1749-8104-6-29 PMC3135491

[30] Rétaux S, Kano S (2010) Midline signaling and evolution of the forebrain in chordates: a focus on the lamprey Hedgehog case. Integrative and Comparative Biology 50: 98–109. 21558191 10.1093/icb/icq032

[31] Mohler J (1988) Requirements for hedgehog, a segmental polarity gene, in patterning larval and adult cuticle of *Drosophila* . Genetics 120: 1061–1072. 3147217 10.1093/genetics/120.4.1061 PMC1203569

[32] Andersson ER, Prakash N, Cajanek L, Minina E, Bryja V, et al (2008) *Wnt5a* regulates ventral midbrain morphogenesis and the development of A9–A10 dopaminergic cells *In Vivo* . PLoS ONE 3: e3517. 18953410 10.1371/journal.pone.0003517 PMC2568809

[33] Zerbino DR, Birney E (2008) Velvet: algorithms for *de novo* short read assembly using de Bruijn graphs. Genome Research 18: 821–829. 18349386 10.1101/gr.074492.107 PMC2336801

[34] Schulz MH, Zerbino DR, Vingron M, Birney E (2012) Oases: Robust *de novo* RNA-seq assembly across the dynamic range of expression levels. Bioinformatics 28: 1086–1092. 22368243 10.1093/bioinformatics/bts094 PMC3324515

[35] Götz S, Garcia-Gomez JM, Terol J, Williams TD, Nagaraj SH, et al (2008) High-throughput functional annotation and data mining with the Blast2GO suite. Nucleic Acids Research 36: 3420–3435. 18445632 10.1093/nar/gkn176 PMC2425479

[36] Conesa A, Götz S, Garcia-Gomez JM, Terol J, Talon M, et al (2005) Blast2GO: a universal tool for annotation, visualization and analysis in functional genomics research. Bioinformatics 21: 3674–3676. 16081474 10.1093/bioinformatics/bti610

[37] Ashburner M, Ball CA, Blake JA, Botstein D, Butler H, et al (2000) Gene ontology: tool for the unification of biology. The Gene Ontology Consortium. Nature Genetics 25: 25–29. 10802651 10.1038/75556 PMC3037419

[38] Consortium TGO (2008) The Gene Ontology project in 2008. Nucleic Acids Research 36: D440–444. 17984083 10.1093/nar/gkm883 PMC2238979

[39] Kanehisa M, Goto S, Hattori M, Aoki-Kinoshita KF, Itoh M, et al (2006) From genomics to chemical genomics: new developments in KEGG. Nucleic Acids Research 34: D354–357. 16381885 10.1093/nar/gkj102 PMC1347464

[40] Kanehisa M, Araki M, Goto S, Hattori M, Hirakawa M, et al (2008) KEGG for linking genomes to life and the environment. Nucleic Acids Research 36: D480–484. 18077471 10.1093/nar/gkm882 PMC2238879

[41] Thompson JD, Higgins DG, Gibson TJ (1994) CLUSTAL W: improving the sensitivity of progressive multiple sequence alignment through sequence weighting, positions-specific gap penalties and weight matrix choice. Nucleic Acids Research 22: 4673–4680. 7984417 10.1093/nar/22.22.4673 PMC308517

[42] Tamura K, Dudley J, Nei M, Kumar S (2007) MEGA4: Molecular Evolutionary Genetics Analysis (MEGA) software version 4.0. Molecular Biology and Evolution 24: 1596–1599. 17488738 10.1093/molbev/msm092

