# Supplementary material for: Post-Embryonic Transcriptomes of the Prawn Macrobrachium rosenbergii: Multigenic Succession through Metamorphosis
Source: PLoS One. 2013 Jan 25;8(1):e55322. doi: 10.1371/journal.pone.0055322 (PMC3555924; doi:10.1371/journal.pone.0055322)

Transcriptome profile:

Distribution of contigs length (A) and normalized coverage distribution (B).

**A**


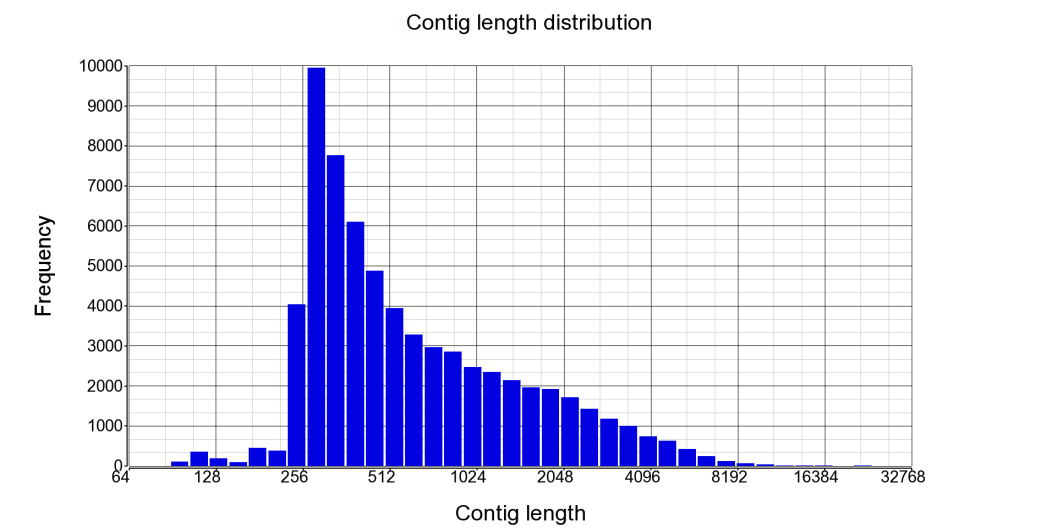


**B**


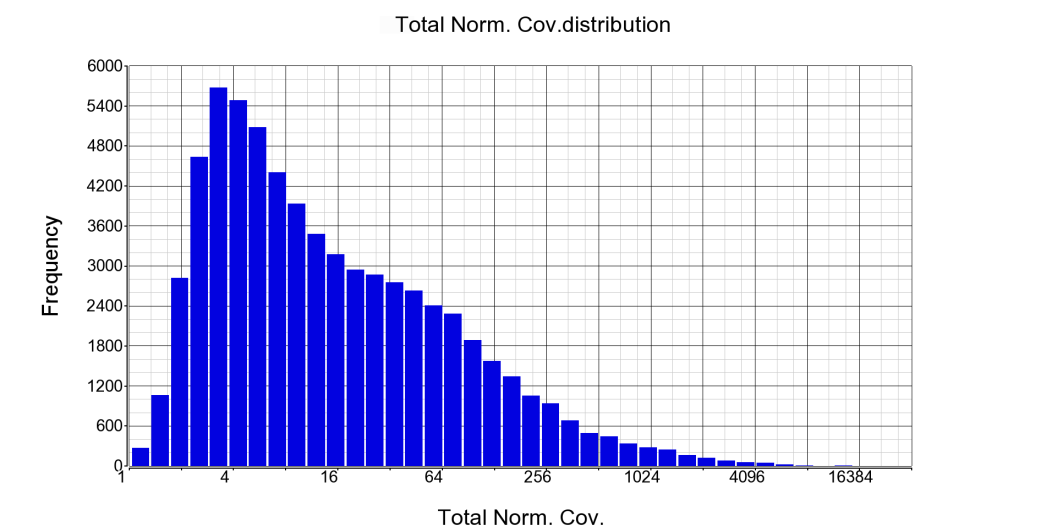

Supplement: Figure S1 — Transcriptome profile. Distribution of contigs length (A) and normalized coverage (B). (DOCX) [file pone.0055322.s001.docx]
