# Supplementary material for: Post-Embryonic Transcriptomes of the Prawn Macrobrachium rosenbergii: Multigenic Succession through Metamorphosis
Source: PLoS One. 2013 Jan 25;8(1):e55322. doi: 10.1371/journal.pone.0055322 (PMC3555924; doi:10.1371/journal.pone.0055322)
Supplement: Figure S4 — Bioinformatics characterization of four transcripts found to be correlated with metamorphosis. Partial transcript and deduced open reading frame is given for Mr-Patched, Mr-Wnt5, Mr-Fz and Mr-CYP15A1. Phylogenetic trees were generated by Mega 4.0 (alignment by ClustalW, maximum parsimony tree 500 bootstraps) for all four with representative orthologs from different taxonomic groups. SMART domain search in Mr-Patched yielded a signal peptide (red) and a Patched domain (grey) with high probability (e−80) and in Mr-Fz it yielded a signal peptide (red) and two Frizzled domains (orange and grey) with high probability (e−66). (DOCX) [file pone.0055322.s004.docx]

***Mr-Patched* partial transcript and deduced ORF (853 amino acids).**

1 C M I T T T L E D V F E S H G R F V A A H P F T V

1 GCTGTATGATCACCACGACTCTGGAGGATGTGTTTGAATCCCACGGGCGTTTTGTGGCAGCTCACCCCTTCACAG

26 V G L C L F I T S V C S L G I M N F E T E L R P F

76 TTGTTGGTCTCTGCTTGTTCATCACATCAGTATGTAGTCTGGGAATAATGAATTTTGAAACAGAGCTACGGCCTT

51 R L W I P Q H S E F I K V L N W Q A E N F P S S Y

151 TTCGTCTCTGGATTCCACAGCATTCGGAGTTTATCAAGGTTTTAAACTGGCAGGCAGAAAATTTCCCTAGTTCTT

76 R R Q I A I W E A D N V L T A R V M Q E M L R L H

226 ACCGTCGGCAAATAGCCATCTGGGAAGCAGACAACGTCCTAACAGCTCGTGTGATGCAGGAAATGCTAAGGCTTC

101 N R V K D T T V G P N A T S W E D L C V R V P S M

301 ACAACAGGGTGAAAGATACCACAGTAGGACCCAATGCTACTTCTTGGGAGGATTTGTGTGTGCGTGTCCCATCCA

126 P M N S H T A T E E E E E E V D Y D S F D Y G S L

376 TGCCCATGAATAGCCACACAGCAACTGAAGAAGAAGAGGAGGAGGTTGATTATGACAGCTTTGATTATGGAAGTT

151 V R K R R S S I T E D E A S D L S L V L P R D Q Y

451 TAGTTCGCAAGCGTCGTAGCAGCATCACAGAAGACGAGGCCTCCGATCTCAGTCTAGTGCTTCCCAGGGATCAGT

176 C R I L E S M P S I C L E T S L L E I W G L D E D

526 ACTGTCGGATTTTAGAGAGCATGCCTAGCATATGCCTTGAGACAAGTCTTTTGGAAATATGGGGGTTAGATGAAG

201 V I M N L T D E Q V V K D I N E V K L S G V F G F

601 ATGTGATTATGAACCTTACGGATGAACAAGTGGTCAAAGATATAAATGAGGTCAAGCTGAGTGGTGTGTTTGGTT

226 R S D F T K Y L G S I T Y D E H G R V V S A K A A

676 TTCGGTCAGATTTTACCAAGTATCTAGGATCAATCACTTATGATGAACATGGGCGTGTGGTTTCAGCAAAGGCTG

251 T H M W I T L V D D Q A I K K G D F V V D K G S G

751 CAACTCACATGTGGATAACTCTTGTTGATGATCAGGCCATCAAGAAAGGAGATTTTGTTGTTGACAAAGGATCTG

276 E P V D A A G M A W E R A W I H S V L E V S S G H

826 GAGAGCCAGTTGACGCAGCTGGTATGGCCTGGGAACGAGCCTGGATTCATTCGGTTTTAGAAGTTTCTTCGGGGC

301 G D I T V Y A Q A A S S F G E I S D D N I W G D V

901 ATGGAGATATCACAGTGTACGCGCAAGCAGCAAGCAGTTTTGGTGAAATAAGCGACGATAACATCTGGGGTGATG

326 K W L V C G I C V M I S F V N M T L G K R N L V Q

976 TCAAATGGCTGGTATGTGGGATTTGTGTCATGATTTCTTTTGTCAATATGACTCTTGGGAAAAGAAATCTTGTTC

351 Q R P L L A F M G I V S V G Q S V A I A Y G L C S

1051 AACAACGTCCGTTGCTAGCTTTCATGGGAATAGTGAGCGTTGGTCAAAGTGTTGCCATTGCTTATGGTCTATGTT

376 M F G I P Y T P V N S I L P I L L I G L G V D D M

1126 CTATGTTTGGAATACCTTATACCCCAGTTAATTCTATACTGCCCATTCTCTTAATTGGACTAGGTGTAGATGATA

401 F V I M A A W E S A G N H K G A S A D L V E R A A

1201 TGTTCGTTATTATGGCAGCATGGGAATCAGCTGGGAACCACAAAGGAGCATCGGCAGATCTTGTTGAACGCGCTG

426 K T M R H A G V A I T V T S L T D V T A F A V G A

1276 CAAAAACAATGCGCCATGCAGGTGTAGCCATTACAGTCACTTCTTTGACTGATGTCACTGCTTTTGCCGTTGGAG

451 S T D L P A L R S F C V Y A S V G I F A V Y V L Q

1351 CTTCTACTGACCTTCCAGCATTGCGTTCCTTCTGCGTTTATGCTTCTGTTGGCATCTTTGCAGTGTATGTACTAC

476 A T F F L V W L V I D Q K R L E G N R N G F F W C

1426 AGGCTACGTTCTTTTTGGTGTGGTTGGTGATAGATCAAAAGAGACTGGAAGGAAACCGCAATGGATTCTTTTGGT

501 I V H R D W K P W A C S Q R D L M A D A F K C L T

1501 GCATTGTGCACCGTGACTGGAAGCCATGGGCATGCAGCCAAAGGGACTTGATGGCAGATGCTTTCAAATGCTTGA

526 K C L L S M Q A R I F I L I F T G S L M A I S I L

1576 CCAAATGTCTACTGAGTATGCAAGCTCGCATATTTATCCTGATTTTCACAGGCTCCCTCATGGCAATTTCAATTT

551 T T L N L H Q E F N P M W F I P Q E S Y L Y K S F

1651 TGACAACACTGAATCTGCACCAAGAATTCAATCCTATGTGGTTTATCCCTCAAGAGTCTTACTTGTACAAGTCAT

576 E A M K L H F P E N G E K G Y I Y F S N I T L P D

1726 TTGAAGCTATGAAGTTACATTTCCCGGAAAATGGTGAAAAGGGATATATATACTTTTCCAATATTACGTTGCCTG

601 D L V H L T K L V D D L K S S G V V T D V S A W F

1801 ATGACTTAGTTCACCTAACTAAGTTAGTTGATGATCTTAAATCTAGTGGAGTGGTAACAGATGTTAGTGCCTGGT

626 T A L D S Y L S H I P E M D E A T M D Y S L L Q D

1876 TCACTGCACTTGACAGTTACTTATCTCACATCCCAGAAATGGACGAAGCGACAATGGATTATTCATTGCTTCAAG

651 K L S I F L Q S S S G A S Y R N D F A V N G Q L E

1951 ACAAGCTTTCAATTTTCCTTCAGTCATCCTCAGGAGCCTCATATCGCAACGACTTTGCTGTTAACGGTCAACTTG

676 C L S P A P P V S S F R I S I T H K P A P S P K E

2026 AATGTCTCTCTCCGGCCCCTCCTGTGTCATCCTTTAGAATTTCGATCACACACAAACCTGCGCCTTCACCTAAAG

701 Q S V A L D T V K S L V A G V P V Q G Y R A A W A

2101 AACAATCTGTGGCACTGGATACAGTCAAGTCGCTGGTAGCTGGTGTACCAGTTCAAGGGTACAGAGCTGCATGGG

726 Q A Y S I W E T N V V V G A E L S R N I F L A G A

2176 CGCAGGCTTATAGCATCTGGGAAACAAATGTAGTTGTCGGCGCTGAATTGAGCAGAAACATATTTCTGGCTGGTG

751 V V G V V T L I L L A S L W A S I L V L V C V A S

2251 CTGTAGTAGGTGTCGTCACACTTATTCTCCTAGCGTCGCTTTGGGCTTCCATTCTTGTCCTGGTATGTGTTGCAT

776 T V I G V C G T M W L W G L T I D T V S C I A L V

2326 CGACAGTTATCGGCGTTTGTGGTACTATGTGGCTTTGGGGCCTCACCATCGATACAGTTTCCTGTATTGCCCTTG

801 L A I G M S V D Y A A H I A H A F L A V K G T N N

2401 TACTGGCAATAGGCATGAGCGTTGATTATGCAGCACACATAGCCCACGCTTTCCTTGCTGTTAAAGGAACTAATA

826 R K E R A R I A L E G V G S A V L Q G G V S T L L

2476 ACAGAAAAGAACGCGCTCGTATTGCGTTGGAAGGGGTTGGTTCTGCTGTGCTTCAAGGAGGAGTGTCCACACTGC

851 A F V

2551 TCGCATTCGTT

**Phylogenetic tree generated by Mega 4.0** (alignment by ClustalW, maximum parsimony tree 500 bootstraps:

**Mr-Patched does not cluster with Patched proteins from other species:** *Apis florae* (380023506); *Bombus impatiens* (350407209); *Megachile rotundata* (383861801); *Aedes aegypti* (157137639); *Culex quinquefasciatus* (170053223).

**Nevertheless, SMART domain search in Mr-Patched yields a signal peptide (red) and a Patched domain (grey) with high probability (e^-80^).**

**
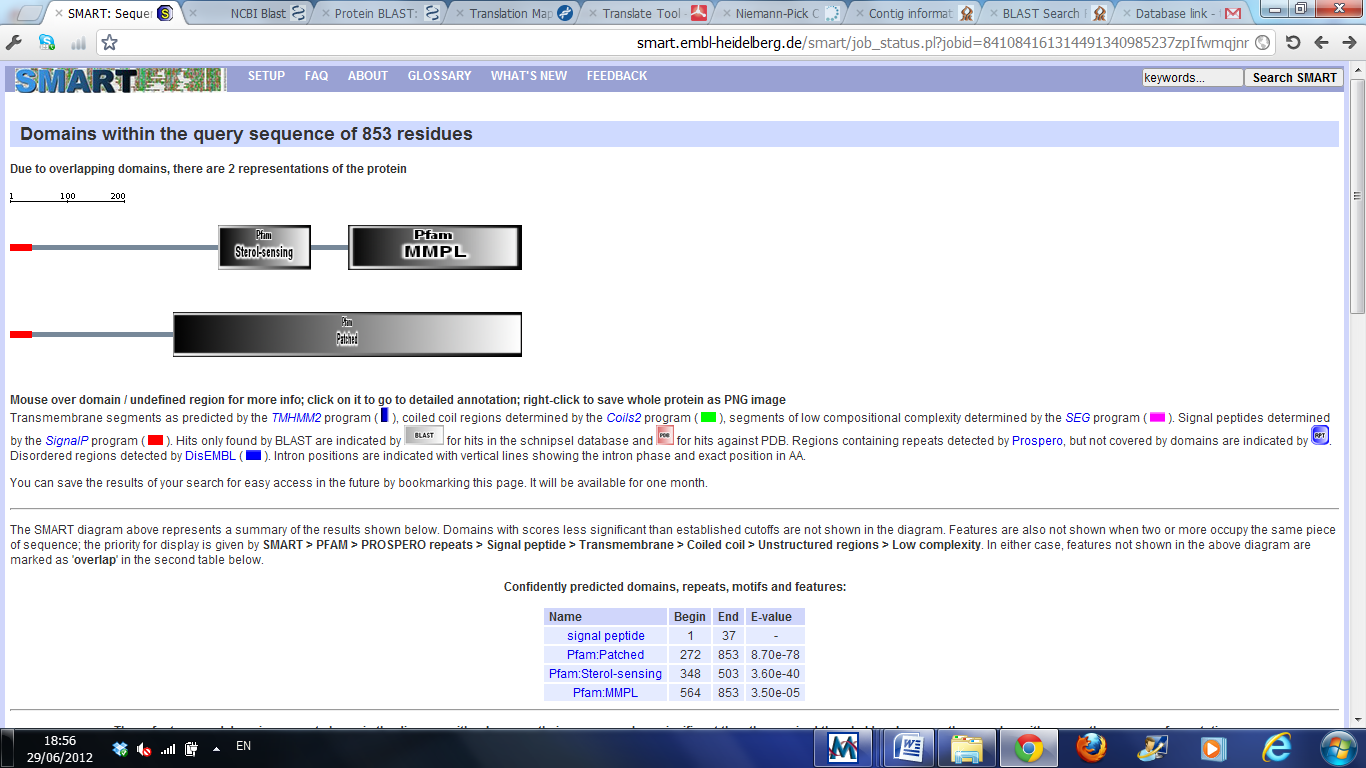
**

***Mr-Wnt5* partial transcript and deduced ORF 286 amino acids.**

1 R G A K T G I H E C Q W Q F R H R R W N C S T V D

1 AGAGGCGCCAAGACAGGCATCCACGAGTGCCAGTGGCAGTTCAGACACAGACGCTGGAACTGTTCGACTGTCGAC

26 D S T V F G P V L Q I P S R E A A F A H A I A S A

76 GATTCCACCGTCTTCGGTCCAGTCTTACAGATACCAAGTCGCGAAGCAGCATTCGCCCACGCCATAGCCTCCGCA

51 G V V D S V S R S C R D G K L T T C G C S R A R R

151 GGGGTGGTGGACAGCGTCTCACGCTCCTGCAGGGACGGGAAGCTGACGACCTGCGGGTGTTCCCGGGCCCGTCGT

76 P R D L N K E W I W G G C G D N V E Y G Y K F T Q

226 CCGAGGGACCTCAATAAGGAGTGGATCTGGGGCGGATGTGGAGACAACGTGGAATATGGATACAAGTTTACCCAG

101 G F V D V R E R E K N Y K R G T I K Q G R Q L M N

301 GGCTTCGTAGATGTGCGCGAGAGAGAGAAAAATTATAAGCGGGGAACGATCAAACAAGGCCGCCAGTTGATGAAT

126 L H N N E A G R R A V I R K T R V T C K C H G V S

376 CTACATAATAATGAAGCAGGAAGAAGGGCAGTAATCCGCAAGACAAGAGTCACCTGCAAATGCCACGGTGTTTCT

151 G S C S L I T C W Q Q L A P F R E V G D L L K E K

451 GGGTCGTGCTCTCTCATTACCTGCTGGCAGCAACTAGCACCTTTCAGGGAAGTAGGCGACTTACTGAAAGAGAAG

176 Y D G A T E V K I N R R G K L Q V K H P Q F N V P

526 TACGATGGTGCTACAGAAGTAAAAATAAACCGCCGTGGGAAACTGCAGGTCAAGCACCCTCAGTTTAATGTCCCT

201 T A E D L V Y L S D S P D Y C R R N N T V G S L G

601 ACAGCTGAAGATCTGGTTTACCTGAGTGACTCTCCAGACTACTGCAGACGCAACAATACTGTAGGGTCTCTGGGA

226 T V D R I C N K T S Q G M D G C G L L C C G R G Y

676 ACCGTCGACCGAATTTGTAATAAAACATCCCAAGGAATGGATGGATGCGGGTTGCTGTGCTGTGGCCGAGGTTAC

251 N S Q K V T L K E R C H C K F H W C C F V E C K T

751 AACTCCCAGAAAGTGACCCTCAAAGAGCGATGTCACTGCAAGTTCCACTGGTGTTGCTTCGTGGAATGCAAAACT

276 C T R V V D L H T C K *

826 TGTACGAGAGTGGTGGACCTCCACACTTGCAAATAATCATGCTGTTGTCTCGACTTAAGGAAAGTGGAGAAACAA

901 AATAAAAAAAAAACTATTCTGCTGTAAAAATCTAAGCTGAGGGTTGAGAAAATGAAAATGCTGCTCTGTAAAACT

976 AGCGCTGTGATACAGAAAAAGAAAGTACGATTCAGCTGCTAAAATTCTGATTTGGAGAGACATTGGTGCAACAGC

1051 ATGAGAGTCGGCAAATTCAGAAACATTGCATAGAGAAAATAAGTACTTAATGTAGTGAAAACTTTTACGAAATCA

1126 GTTGATGCTGTCAGTGATTAACTATGACTACTGCATGTAGTGAAAAAGACACCGTTTAAGAATAGTAGTGATAAT

1201 GAATACTCGTTAAATATTTGTACTAAAATTGGCCAAGATTTCATGCAATGTCCCCCTGTAGAAATGTGAATTACC

1276 ACTACAGTTAGAGCTGTGCACGTACCAACATTTTCATTTTGTGCAATGTCATGAGAAAAATTGACATTTGCAGGA

1351 GATTATTTATGCACTTAACCTTAAGCCTGTATAGGACTCCCTTCTTATTAAAGCAATTTCATTAATGTAAGCGAT

1426 GTACAGTCTCCAGGTGTTAACTTTACATAATATATTAAATATTTGTATTGTCAAGGTGTTTAGTGGCAGGTTTTT

1501 TTTATTTTTCAGTGT

**Phylogenetic tree generated by Mega 4.0** (alignment by ClustalW, maximum parsimony tree 500 bootstraps:

Mr-Wnt5 clusters with Wnt5 of *Daphnia pulex* anf Wnt5b of *Cupiennius salei* but not with Wnt1.

***Mr-Fz* partial transcript and deduced ORF of 610 amino acids.**

1 TTGTGGAAGAAGAAAGATATGTGTCGTTGTGTCCCTGTCGCGTGTGTGGTACTATCGAATATTTGATGATGAATG

76 AGTTATTCTATATGATCGAATGATTTAACGAAAGCAGAATTGTGTTTGACGATTAAGGAAAACGGAAGCGTGTGA

1 M A R R A A G K F G G G E R

151 AAAACGTGGAATATTGGAGTTTGAATACACAGCATGGCGAGAAGGGCAGCGGGGAAGTTTGGAGGTGGCGAGCGG

76 V T A A A S S S P Y V S C C R L L F L L V L L I T

226 GTAACTGCTGCTGCCTCTTCTTCCCCTTACGTTAGCTGTTGCAGATTGCTTTTCCTTTTAGTGTTGTTGATTACA

101 V C S A Q I P P V G G G A G G I P I E G G I R L G

301 GTTTGTAGTGCACAAATTCCCCCGGTTGGAGGGGGCGCTGGAGGTATTCCCATCGAAGGAGGAATTCGCCTGGGA

126 G G H G R V E H I S D H D L L P R H N R C E P I T

376 GGAGGGCATGGCCGTGTGGAACATATCTCTGACCACGACCTCTTACCTCGGCATAATCGGTGTGAACCCATTACC

151 I T L C K D L Q Y N T T I M P N L L K H H T Q E E

451 ATCACTTTGTGCAAAGATCTCCAGTATAACACCACCATCATGCCCAACCTTCTGAAACATCATACCCAGGAAGAG

176 A G M E V H Q F F P L V K V K C S G D L Q F F L C

526 GCTGGGATGGAAGTCCATCAGTTTTTTCCTTTGGTCAAAGTGAAGTGCTCGGGCGACCTACAGTTTTTCTTGTGC

201 S V Y V P V C T I M E R P L P P C R H L C L S A K

601 AGTGTTTACGTACCCGTGTGCACCATCATGGAAAGGCCTCTACCTCCTTGCCGACACTTGTGTCTTTCTGCCAAA

226 D G C E D L M N K F G F Q W P E S L D C N K F P A

676 GATGGATGTGAAGACCTCATGAACAAGTTTGGTTTCCAGTGGCCTGAATCCCTCGATTGCAACAAGTTCCCGGCA

251 G P H E E L C V G E N N T A P H D P P P S F P E P

751 GGACCCCACGAAGAACTTTGCGTAGGAGAGAACAACACGGCCCCCCATGACCCTCCCCCTTCCTTTCCGGAGCCT

276 R P P V G G T Q Y A G K A F Q C P A H F K V P K R

826 AGACCTCCTGTAGGGGGAACACAATATGCTGGCAAGGCTTTTCAGTGCCCTGCCCACTTCAAAGTGCCCAAGAGA

301 L E Y K L R V G T V E A P D C G A P C N G M F F N

901 CTCGAGTACAAATTGAGAGTAGGAACCGTAGAGGCCCCTGACTGCGGTGCCCCTTGCAATGGCATGTTCTTTAAT

326 E E E L N F S R N W V G G W A A V C L A S T T F T

976 GAGGAGGAACTCAACTTTTCAAGAAACTGGGTAGGCGGATGGGCTGCAGTTTGTCTGGCTTCAACCACCTTCACC

351 I A S F L A D V R R F R Y P E R P I I F I S M C Y

1051 ATCGCATCCTTTTTGGCAGATGTCCGCCGTTTTCGGTACCCCGAGCGCCCGATCATCTTCATCAGCATGTGTTAC

376 W F I A A T Y V V G L F Q G D K I A C D E P W E P

1126 TGGTTTATCGCCGCCACTTACGTGGTGGGGCTCTTCCAGGGCGACAAAATCGCCTGCGACGAGCCCTGGGAACCT

401 P Q Y L P E L R E H M V R T I T Q G V D R E W C T

1201 CCGCAATATTTGCCCGAACTGAGGGAGCACATGGTGAGGACCATCACCCAGGGGGTCGACAGGGAATGGTGCACC

426 I V F M T L Y F F S M A A S I W W V V L T L T W F

1276 ATTGTCTTCATGACCCTCTATTTCTTTTCGATGGCTGCCTCAATTTGGTGGGTGGTGCTCACCCTCACCTGGTTC

451 L A A G L K W S H E A I E N N S A W F H L A A W A

1351 TTAGCTGCAGGTCTCAAGTGGAGCCACGAAGCCATTGAGAACAACAGTGCCTGGTTTCATCTGGCAGCATGGGCC

476 I P A V K T I V I L A T E N V E G D V M T G V C F

1426 ATCCCCGCCGTCAAGACCATCGTCATTTTGGCCACCGAAAATGTTGAAGGTGACGTGATGACGGGGGTGTGTTTC

501 V G L W N V R A L Q G F V L A P L F F Y L V M G T

1501 GTGGGCCTGTGGAATGTACGAGCGCTTCAGGGGTTTGTATTGGCACCGTTGTTCTTCTACTTGGTCATGGGGACT

526 I F L L T G F V S L F R I R T I M K H D G T K T D

1576 ATCTTCCTGCTGACCGGTTTCGTCTCGCTCTTCCGCATCAGGACCATCATGAAGCACGACGGCACGAAGACAGAC

551 K L E R F M V R I G V F S V L Y T V P A T V L V A

1651 AAGCTGGAGAGGTTCATGGTGCGCATTGGAGTGTTCAGCGTCCTTTACACCGTGCCAGCGACCGTCCTGGTGGCT

576 C L F Y E Q A Y H D E W M V A W Q R D K C Q I R D

1726 TGTCTTTTCTACGAACAGGCCTATCACGACGAATGGATGGTGGCGTGGCAGAGGGATAAGTGCCAGATCCGCGAC

601 E P W V S Y T I N C P P N V D H D T P L S K P D F

1801 GAGCCCTGGGTCTCCTACACCATAAACTGCCCTCCCAATGTCGATCACGACACCCCCTTGTCCAAGCCCGACTTT

626 I F F M I K Y L S T L V V G I T S G F W V W S G K

1876 ATTTTCTTCATGATCAAGTACCTGAGCACCCTGGTGGTGGGCATCACTTCAGGCTTCTGGGTATGGTCTGGCAAA

651 T L A V W K N C Y N R C F G R R T E S Y V *

1951 ACGCTCGCTGTCTGGAAGAACTGTTACAACCGATGCTTCGGACGACGCACCGAAAGCTATGTGTGACAATCCGCC

2026 CCCGCCTGGCCTAATAATGCTCACGCCCTAACTCAGGGGGGCGCATCCGCTCCTTTAACCCCTCAGCCTCCTCCA

2101 TCCACTGCCACAACTACAGCCGTAGCTCGGCTGTTACCTGAAGAGGCTGTGCCCTTCAGACCCACACACACTCCT

2176 GAGGAGACTAACTTCTAAAGGAGTCAGGTTTCCCGAAGGAGGGAGGAGGATCTTCAGGGATTGGGGCGAGTGGAT

2251 ACGAACACTTCCTTGGAGAAGAGGAAGGGATAAGTACACTATCTGCACTTAGAAGTCTATTACAACACCAGTAAT

2326 AATAATAATAATAATAATACTACAGAGTAAAAGAAGAGTAATAGCAATGTACTATAGCACTTATTCAGATGTGTT

2401 TTGTATTGCTGCCAATGTTTTTGTAAAGTTGCCAGGTAACACTTTATCTGTTAATTAATTATAAAAGGAACAGGT

2476 GCAGGTTGTGCATGTTCAGTATCCAATTTGAGCTGCTTTAGATCTTTCATAGAATGAAATTCTATTTTAATGTAA

2551 CATTTCAGATCGAAACTTGAATAAAGGTCTTTTAAGCCAGGCGGGAAATTATATATATAAATATATATCATTAAC

2626 AATGACGAACTTTTTAGTTTTATCGCAATAAACTTTGTACTTCCTATGACAGGAGGATATTTATTTTAGTTCTTG

2701 TCCTTATGCATTTCTAGTTTTTGTTGACTACTGGTGCATCTTTTGGTGCAATGTAAATCTCCCGATCAGGATCGT

2776 ATGTATCAGTGAAAATGTACATCTGGAAAAAAAGGGGGTCGAGTGAATAATAATAATAATAATA

**Phylogenetic tree generated by Mega 4.0** (alignment by ClustalW, maximum parsimony tree 500 bootstraps:

**Mr-Fz clusters with insects frizzled proteins:** *Apis mellifera* (66505413); *Bombus impatiens* (350401487); *Ixodes scapularis* (241998672); *Danio rerio* (25092729); *Xenopus laevis* (147902240).

**SMART domain search in Mr-Fz yields a signal peptide (red) and two Frizzled domains (orange and grey) with high probability (e^-66^).**


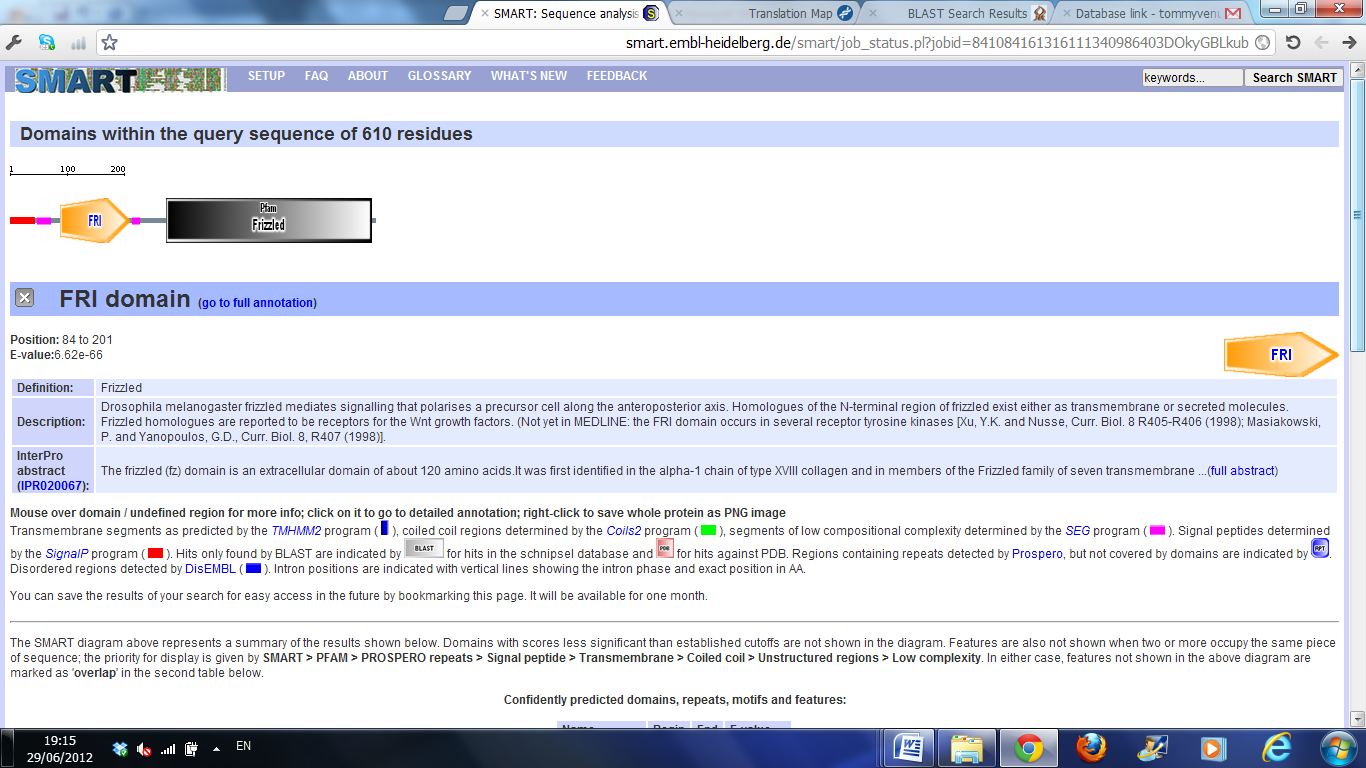


***Mr-CYP15A1* partial transcript and deduced ORF of 472 amino acids.**

1 L L Y Y N S R K P A G F P P G P P R Y P

1 CCTTGCTATACTATAATAGCAGAAAGCCAGCGGGATTTCCACCAGGACCGCCTCGGTACC

21 F I G Y P F L E T H L L H K Q L W R L S

61 CGTTCATCGGCTACCCGTTCCTCGAGACGCACCTGCTCCACAAGCAACTGTGGCGCCTCT

41 D T Y G P V V G I Y L G P Q R T V I V N

121 CTGATACCTACGGACCGGTCGTCGGCATTTACTTGGGTCCGCAACGGACCGTCATCGTCA

61 G W E A V K E A L T N D D L N G R P E N

181 ACGGATGGGAGGCCGTTAAAGAGGCCCTGACCAACGACGACCTCAACGGACGTCCGGAGA

81 F F M R F R D G G K F K G V I F T E G E

241 ATTTCTTCATGAGGTTTCGAGACGGAGGGAAGTTCAAAGGCGTCATATTCACCGAGGGAG

101 L W K E Q R R F S L H N F R N L G F G K

301 AACTTTGGAAGGAGCAGCGCAGGTTCTCCCTCCACAACTTCCGCAATTTGGGATTCGGGA

121 R S H E A V I L E E V Q E L I K E V E E

361 AGCGCTCCCACGAGGCCGTCATACTCGAGGAAGTCCAGGAGCTCATCAAAGAAGTAGAGG

141 T D G S V E F Q V K L G V S A T N I L W

421 AAACTGATGGAAGCGTCGAATTTCAGGTGAAGCTGGGTGTGTCCGCGACCAACATCCTTT

161 A L M G G T R F R R N D P R L I D L V K

481 GGGCACTCATGGGAGGAACCAGGTTCCGCAGGAATGACCCGAGGCTGATCGATTTGGTCA

181 S L D R A F R A G E V A G S I V D M V P

541 AGAGCCTGGACAGAGCTTTCAGGGCTGGGGAGGTGGCTGGATCCATCGTGGACATGGTCC

201 A V R H F L P K D S K F S T V M D G L C

601 CTGCAGTTCGTCACTTCTTGCCAAAGGACTCCAAATTTAGCACTGTCATGGATGGCTTGT

221 K V K N F I A E T V E E H K A T L D T N

661 GTAAGGTTAAAAATTTCATTGCGGAAACAGTGGAAGAGCACAAAGCAACGCTGGACACAA

241 S P R D F I D I F L T E M Q K Q K S N E

721 ACAGCCCAAGAGATTTCATCGACATCTTCTTGACTGAGATGCAGAAGCAAAAAAGCAATG

261 D T T F T E D Q L L A L C S D M F M A G

781 AGGATACAACATTCACTGAGGACCAGCTCTTGGCACTGTGTTCCGACATGTTCATGGCGG

281 S E T G S S S V S F V L L F C A L Y P D

841 GGTCAGAGACTGGTTCATCTTCGGTGTCCTTCGTGCTACTGTTTTGCGCTCTCTATCCAG

301 V M N K I H K E L D S V V G Q D R L P S

901 ACGTCATGAACAAGATCCACAAAGAGTTGGACTCCGTCGTGGGTCAAGACCGCCTTCCTT

321 L D D R P K L V Y T D A V L T E M A R I

961 CGCTGGACGATCGTCCGAAGTTAGTCTATACCGATGCTGTGTTAACTGAAATGGCTAGGA

341 R G V A P L T A P H C A M R D T K L Q G

1021 TACGTGGCGTCGCACCGCTCACCGCGCCCCACTGCGCTATGAGGGACACCAAGCTTCAGG

361 Y N I P E R T T V V V N L Y S I L M D K

1081 GATACAACATTCCTGAGCGGACGACTGTGGTGGTGAATCTGTACAGCATCCTGATGGACA

381 D Y W G D P E I F R P E R F L N P D G S

1141 AGGATTATTGGGGTGACCCAGAGATCTTCCGGCCCGAACGGTTCCTGAATCCGGACGGGT

401 L R K D E R L I P F G K G R R S C L G E

1201 CCCTGAGGAAGGACGAACGCTTGATTCCTTTTGGAAAAGGACGACGGTCGTGTTTGGGAG

421 S L A R M S L F L I F T G L M Q K F S F

1261 AGTCCTTGGCCCGCATGTCGCTGTTCCTCATCTTCACGGGCTTGATGCAGAAGTTTTCCT

441 T I D P S V P F P D T E G R G G L S L C

1321 TCACAATCGACCCTTCTGTCCCCTTCCCAGACACCGAGGGCAGAGGAGGACTCTCTCTGT

461 P P R F K V F A K S R F *

1381 GCCCTCCAAGGTTCAAGGTCTTTGCCAAGAGCAGATTCTGATGAGGAGGACGCCGGCATT

1441 TCTTCCTCCAATATTTCCCCTCTTCCAGGATTCTTTTCCAGGATCTTTTCCAGGATTCCT

1501 GGTCCTACAGTCTACTGTCTTTCGAAGGCGTATATATCATATCCTTCGTAGGAATCATTT

1561 CTCTTTTTTTTTTTGTCTGTTAAAACCTCCAATACTTCCCATTTTCCAGGATTTCTGCTC

1621 CTGTAGCTCTGTGGCTTCCGAAGGGAATGTATATCATATCCTTCGCAGGAGTCATCTTTT

1681 TTTGTTTGTTTGTTAGAAAATTGTGACATGGCTGGATAGTGACGTCAGGTTTTCCCCTTC

1741 CGACGGCCTCTGTGTTTAAGTATTGTTAATGTTTATTTCAAATGTTTACCTGTAGTGTTT

1801 TATCGAGAATTCGATATTAAATATTAAACGCTTGATCAATATGTGGAATATTAGCTGTAA

1861 AAATATTAGTGGATATATACTCATGAGAAAGTTGGTTTTAATATCAAAGAAATCTATTCA

1921 GTCATCGTATCTTCCCAGTAATTTGAAATCTACTAGACCTTATTTCATTCAAATTCGTTC

1981 AGACCCAGGCTAGGTAAAGCACGAAGTTGCCCATTCCAAAGGCCTCCGATTGAAAAAAAA

2041 AA

**Phylogenetic tree generated by Mega 4.0** (alignment by ClustalW, maximum parsimony tree 500 bootstraps:

Mr-CYP15A1 clusters with methyl farnesoate epoxidase from *Schistocerca gregaria* (Sg-MF epoxidase), *Tribolium castaneum* (Tc) and *Diploptera punctata* (Dp)CYP15A1 and *Aedes aegypti* (Aa) cytochrome P450 (CYP450). Other CYP450 identified in *Macrobrachium rosenbergii* (Mr) clustered into different branches: Mr-CYP4C39 clustered with three other isotigs (B, C, D) and Mr-CYP2L1A clustered with its isotig Mr-CYP2L1B. *Apis mellifera* cytochrome P450 305a1 (Am-CYP305a1) remotely clustered with CYP15A1 sequences.
